# Diagnostic Performance of [ ^18^ F]FDG PET/MRI and [ ^18^ F]FDG PET/CT in the Detection of Lymph Node Metastases in Colorectal Cancer: A Meta-analysis

**DOI:** 10.1055/s-0045-1812491

**Published:** 2025-11-12

**Authors:** Dapeng Shen, Lexuan Chen, Pengyuan Su, Peng Chen, Wei Zeng, Shen Chen, Jiemiao Shen

**Affiliations:** 1 The Second Clinical Medical College, Nanjing Medical University, Nanjing, People's Republic of China; 2 School of Pediatrics, Nanjing Medical University, Nanjing, People's Republic of China; 3 School of Nursing, Nanjing Medical University, Nanjing, People's Republic of China

**Keywords:** [ ^18^ F]FDG PET/CT, [ ^18^ F]FDG PET/MRI, colorectal cancer, lymph node metastases, meta-analysis

## Abstract

**Background:**

Colorectal cancer (CRC) ranks among the leading causes of cancer-related mortality worldwide. Accurate detection of lymph node metastases plays a crucial role in determining disease stage and guiding treatment decisions. Fluorine-18 fluorodeoxyglucose (18F-FDG) positron emission tomography (PET)/computed tomography (CT) and 18F-FDG PET/magnetic resonance imaging (MRI) are advanced imaging modalities widely used in clinical practice. The study aimed to compare the diagnostic accuracy of [ ^18^ F]FDG PET/CT and [ ^18^ F]FDG PET/MRI in detecting lymph node metastases in CRC patients.

**Methods:**

A comprehensive literature search was conducted across PubMed, Embase, and Web of Science databases to identify relevant studies published up to February 2025. Inclusion criteria encompassed studies evaluating the diagnostic performance of [ ^18^ F]FDG PET/CT and [ ^18^ F]FDG PET/MRI for detecting lymph node metastases in CRC patients. Sensitivity and specificity were analyzed using the DerSimonian and Laird random-effects model, with results adjusted by the Freeman-Tukey double arcsine transformation. The quality of the included studies was appraised using the Quality Assessment of Diagnostic Accuracy Studies (QUADAS-2) tool.

**Results:**

This meta-analysis incorporated 24 studies with a cumulative total of 3,369 patients. The findings revealed that [ ^18^ F]FDG PET/CT exhibited significantly lower sensitivity (0.75 vs. 0.93, *p* = 0.0096) and a lower AUC value (0.81 vs. 0.96) compared with [ ^18^ F]FDG PET/MRI in detecting lymph node metastases among CRC patients. Both [ ^18^ F]FDG PET/CT and [ ^18^ F]FDG PET/MRI demonstrated similar specificity (0.77 vs. 0.88, *p* = 0.1892). Furthermore, the funnel plot asymmetry test indicated no significant publication bias across any of the outcomes (Egger's test: all *p* > 0.05).

**Conclusions:**

Our meta-analysis demonstrated that [ ^18^ F]FDG PET/MRI has higher sensitivity and comparable specificity to [ ^18^ F]FDG PET/CT in detecting lymph node metastases in CRC patients, suggesting its potential superiority for preoperative staging and postoperative surveillance. However, the limited number of direct head-to-head studies (only two) underscores the need for larger, prospective studies to validate these findings and assess their impact on clinical decision-making and patient outcomes.

## Introduction

Colorectal cancer (CRC) is the most prevalent malignancy affecting the gastrointestinal tract, impacting the proximal colon, distal colon, or rectum. It stands as the leading cause of cancer-related deaths globally and ranks third among the most frequently diagnosed cancers worldwide. 1 2 Over recent decades, the incidence of CRC has steadily increased, with ∼1.8 million new cases and over 900,000 deaths reported annually. 3 CRC frequently metastasizes to lymph nodes, significantly worsening prognosis and survival rates. Patients with lymph node involvement often face a poorer prognosis, increased recurrence risk, and reduced overall survival. 4 According to the National Cancer Institute, the regional lymph node spread (stage III) occurs in 36% of cases, with a 5-year relative survival rate of ∼73.4%. 5 Early detection of lymph node metastases and accurate staging play pivotal roles in the effective management of CRC, significantly influencing treatment strategies and patient prognoses. 3 6

Imaging plays an increasingly critical role in the diagnosis, staging, metastasis assessment, and treatment planning of CRC based on prognostic factors. 7 Conventional methods for staging CRC include computed tomography (CT), magnetic resonance imaging (MRI), and biopsy. While these techniques are integral to clinical practice, they have notable limitations. Standard CT scans can only detect lymph nodes larger than 2 cm. Both CT and MRI are limited by their low sensitivity in identifying small metastatic lymph nodes. 8 9 Although biopsy is the gold standard for tissue diagnosis, it is invasive and comes with risks such as bleeding and infection. 10 Moreover, sampling errors and interpretative variability can undermine diagnostic accuracy. 11

The emergence of hybrid imaging techniques, such as fluorine-18 fluorodeoxyglucose (18F-FDG) positron emission tomography (PET) combined with MRI (PET/MRI) or CT (PET/CT), has significantly revolutionized the diagnostic approach to CRC. Recent research indicates that both [ ^18^ F]FDG PET/MRI and [ ^18^ F]FDG PET/CT are highly effective in staging diagnosis of CRC. 12 13 14 15 However, ongoing debate surrounds the comparative diagnostic efficacy of PET/MRI versus PET/CT in detecting lymph node metastases in CRC. Some studies support the superiority of PET/MRI, 16 noting enhanced soft-tissue contrast and reduced radiation exposure, potentially aiding in detecting small or hidden metastases. Conversely, other studies highlight the advantages of PET/CT, including shorter acquisition times and adequate diagnostic accuracy. 17 18 This study specifically focuses on the diagnostic performance analysis of lymph node metastasis, a single type of metastasis, in CRC, whereas previous literature may have addressed a broader range of metastatic types (e.g., distant metastasis or systemic metastasis). 19 By concentrating on lymph node metastasis, this study provides a more in-depth exploration of the sensitivity, specificity, and clinical applicability of two imaging modalities (PET/MRI and PET/CT) in this specific context, thereby minimizing the potential confounding effects of other metastatic types (e.g., liver or bone metastasis) on the evaluation of diagnostic efficacy. Moreover, several studies find no significant overall diagnostic performance between the two modalities, underscoring the necessity for further comparative research to clarify these discrepancies. 20 21

Given the existing controversy and the clinical implications of selecting the optimal imaging modality for CRC, this meta-analysis aims to systematically pool and assess the diagnostic accuracy of [ ^18^ F]FDG PET/MRI and [ ^18^ F]FDG PET/CT in the detection of lymph node metastases in patients with CRC.

## Methods

### Search Strategy

This meta-analysis followed the guidelines of the Preferred Reporting Items for Systematic Reviews and Meta-Analyses of Diagnostic Test Accuracy (PRISMA-DTA). 22 The study protocol was registered with PROSPERO under the registration number CRD2024541527.

A comprehensive literature search was conducted in the PubMed, Embase, and Web of Science databases to find relevant studies published up to February 2025. The search included the following keywords: “Colorectal cancer,” “Lymph node metastasis,” “PET/MRI,” and “PET/CT.” Supplementary Table S1 (available in the online version only) provides detailed search strategies. To ensure thoroughness, the reference lists of all included studies were manually checked for additional pertinent articles.

### Inclusion and Exclusion Criteria

#### Inclusion and Exclusion Criteria

Studies were eligible for inclusion in this meta-analysis if they met the following criteria:

P: Patients diagnosed with CRC and suspected of having lymph node metastases.
I: Studies that utilized [ ^18^ F]FDG PET/MRI for the detection of lymph node metastases.
C: Studies that utilized [ ^18^ F]FDG PET/CT for the detection of lymph node metastases.
O: Studies reporting diagnostic performance metrics, including sensitivity, specificity, true positives, true negatives, false positives, and false negatives.
S: Both retrospective and prospective studies were included.

Studies were excluded if they met any of the following criteria: duplicate publications; abstracts without corresponding full-text articles; editorial comments, letters, case reports, reviews, or meta-analyses; studies with titles and abstracts deemed irrelevant to the research question; non-English full-text articles; or studies lacking complete or clear data necessary for calculating sensitivity or specificity.

### Quality Assessment

Two independent researchers evaluated the quality of the included studies utilizing the QUADAS-2 (Quality Assessment of Diagnostic Accuracy Studies) tool. This assessment framework encompasses four key domains: (1) patient selection, (2) index test, (3) reference standard, and (4) flow and timing. The risk of bias for each domain was classified as “high risk,” “low risk,” or “unclear risk.”

### Data Extraction

Data extraction was independently performed by two researchers. The extracted information included: authorship, publication year, type of imaging technique, study details (country, design, analysis method, and reference standard), patient demographics (total number of patients, clinical indications, mean or median age, and history of previous treatments), and technical aspects (type of scanner, dose of ligand, and method of image analysis). Discrepancies between the researchers were resolved through discussion to reach a consensus, ensuring the accuracy of the data.

### Statistical Analysis

The sensitivity and specificity of the imaging techniques were analyzed using the DerSimonian and Laird random-effects model, with results transformed through the Freeman-Tukey double arcsine method. Confidence intervals (CIs) were determined using the Jackson method. Diagnostic analysis was performed using summary receiver operating characteristic (sROC) curves, with subsequent calculation of the area under the curve (AUC). To assess heterogeneity within and between study groups, Cochrane's Q test and the inconsistency index ( *I ^2^* ) were employed. In cases where significant heterogeneity was identified ( *p* < 0.05 or *I ^2^* > 50%), further sensitivity and meta-regression analyses were conducted to explore potential sources of heterogeneity. Publication bias was assessed using funnel plots and Egger's test. A threshold of *p* < 0.05 was set for statistical significance. Data analysis and graphical representation were performed using R software, version 4.2.3.

## Results

### Study Selection and Data Extraction

**Fig. 1:**
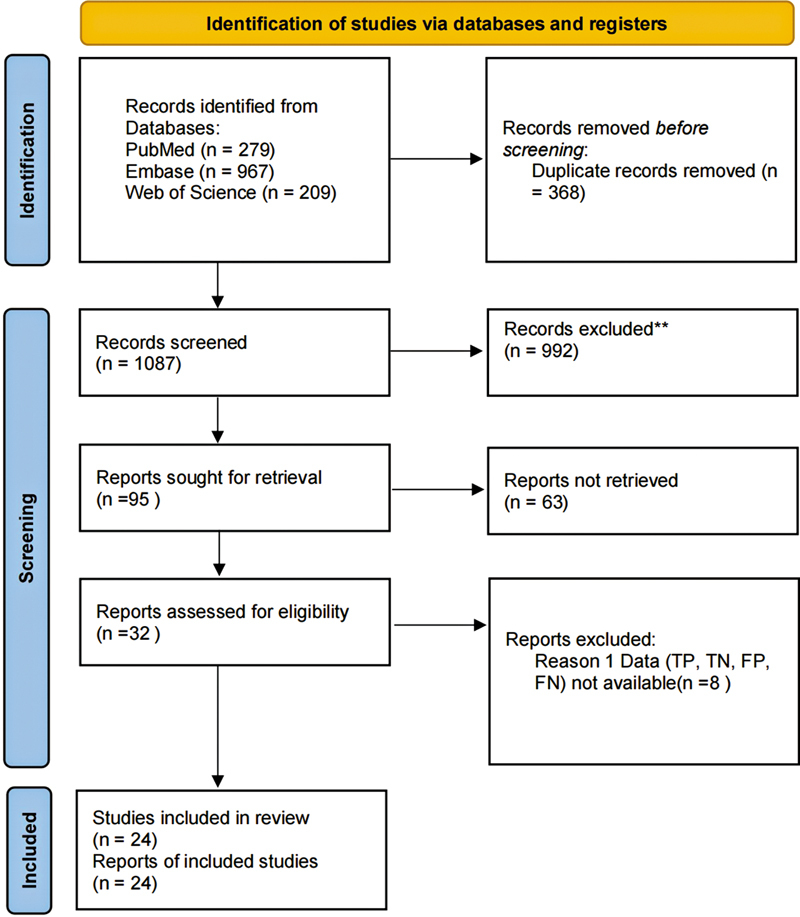
PRISMA flow diagram illustrating the study selection process.

The initial search identified 1,445 publications. After removing 368 duplicates, 1,087 articles were excluded for not meeting the eligibility criteria. A detailed review of the full texts of the remaining 32 articles led to the exclusion of 8 studies due to insufficient data (true positives, false positives, false negatives, and true negatives). Consequently, 24 studies assessing the diagnostic performance of [ ^18^ F]FDG PET/CT and [ ^18^ F]FDG PET/MRI were included in the meta-analysis. 23 24 25 26 27 28 29 30 31 32 33 34 35 36 37 38 39 40 41 42 43 44 45 46 The selection process is depicted in Fig. 1 , following the PRISMA flow diagram.

### Study Description and Quality Assessment

The 20 selected studies encompassed a total of 3,369 patients diagnosed with CRC, with individual study sample sizes ranging from 10 to 509 patients. 23 24 25 26 27 28 29 30 31 32 33 34 35 36 37 38 39 40 41 42 43 44 45 Among these, 19 studies adopted a retrospective approach, 23 24 25 26 27 28 29 31 32 33 34 35 36 37 40 41 42 43 46 while 5 were prospective in design. 30 38 39 44 45 In terms of analysis methods, 22 studies conducted patient-based analyses, 23 24 25 26 27 28 29 30 31 32 33 34 35 36 37 38 39 40 43 44 whereas 2 studies used lesion-based analyses. 41 42 Fourteen studies utilized pathology as the reference standard, 24 25 26 27 28 29 30 32 33 34 35 36 41 42 43 45 7 combined pathology with follow-up imaging, 26 31 37 38 39 40 46 and 3 relied exclusively on follow-up imaging. 23 41 44 Regarding clinical indications, 19 studies focused on patients at the initial diagnosis stage, 23 24 25 26 27 28 29 30 31 32 33 34 35 36 37 41 42 43 45 1 study included patients only after treatment, 38 and 4 studies involved patients at both initial and post-treatment stages. 39 40 44 46 The study and technical characteristics are detailed in Tables 1 and 2 .

**Table 1:** Study and patient characteristics of the included studies for [ ^18^ F]FDG PET/CT

Author	Year	Type of imaging test	Study characteristics				Patient characteristics			
			Country	Study design	Analysis	Reference standard	No. of patients	Clinical indication	Mean/Median age	Previous treatment
Engel et al	2024	[ ^18^ F]FDG PET/CT	Switzerland	Retro	PB	Pathology	471	Initial staging in colorectal cancer	Mean: 69	NA
Nasr et al	2023	[ ^18^ F]FDG PET/CT	Egypt	Retro	PB	Follow-up imaging	79	Initial staging in colorectal cancer	Mean: 57	NA
Gauci et al	2023	[ ^18^ F]FDG PET/CT	Australia	Retro	PB	Pathology	34	Initial staging in colon cancer	Mean: 65	NA
Gunduz et al	2023	[ ^18^ F]FDG PET/CT and [ ^18^ F]FDG PET/MRI	Türkiye	Pro	PB and LB	Follow-up imaging	78	Initial staging in colon cancer	Mean: 58.8	Chemotherapy/radiotherapy/surgery
Xu et al	2023	[ ^18^ F]FDG PET/CT	China	Retro	PB	Pathology	264	Initial staging in colorectal cancer	Mean: 64.69	NA
Yukimoto et al	2022	[ ^18^ F]FDG PET/CT	Japan	Retro	PB	Pathology and follow-up imaging	541	Initial staging in colorectal cancer	Mean (range): 67 (23–92)	NA
Yukimoto et al	2021	[ ^18^ F]FDG PET/CT	Japan	Retro	PB	Pathology	84	Initial staging in rectal cancer	Mean (range): 62 (27–83)	Chemotherapy/radiotherapy
Bae et al	2018	[ ^18^ F]FDG PET/CT	Korea	Retro	PB	Pathology	176	Initial staging in rectal cancer	Mean (range): 62 (27–83)	Chemotherapy/radiotherapy
Chen et al	2018	[ ^18^ F]FDG PET/CT	China	Retro	PB	Pathology	90	Initial staging in colorectal cancer	Mean: 66	NA
Atici et al	2016	[ ^18^ F]FDG PET/CT	Türkiye	Pro	PB	Pathology	61	Initial staging in colorectal cancer	Mean: 59.16	NA
Paspulati et al	2015	[ ^18^ F]FDG PET/CT and [ ^18^ F]FDG PET/MRI	USA	Pro	PB	Pathology	12	Initial staging in colorectal cancer	Mean: 59	NA
Kwak et al	2012	[ ^18^ F]FDG PET/CT	Korea	Retro	PB	Pathology and follow-up imaging	473	Initial staging in colorectal cancer	Mean: 59	Surgery, preoperative FDG-PET/CT
Kim et al	2011	[ ^18^ F]FDG PET/CT	Korea	Retro	PB	Pathology	30	Initial staging in rectal cancer	Mean: 62	NA
Ono et al	2009	[ ^18^ F]FDG PET/CT	Japan	Retro	PB	Pathology	25	Initial staging in colorectal cancer	Mean: 67.3	NA
Akiyoshi et al	2008	[ ^18^ F]FDG PET/CT	Japan	Retro	PB	Pathology	65	Initial staging in colorectal cancer	Mean: 62	Chemotherapy/radiotherapy
Tsunoda et al	2008	[ ^18^ F]FDG PET/CT	Japan	Retro	PB	Pathology	88	Initial staging in colorectal cancer	Mean: 60.6	NA
Seto et al	2022	[ ^18^ F]FDG PET/MRI	Japan	Retro	PB	Pathology	23	Initial staging in rectal cancer	NA	Chemotherapy/radiotherapy
Catalano et al	2021	[ ^18^ F]FDGPET/MRI	USA	Retro	PB	Pathology and follow-up imaging	62	Initial staging in rectal cancer	Mean: 60	NA
Crimì et al	2020	[ ^18^ F]FDG PET/MRI	Italy	Pro	PB	Pathology and follow-up imaging	36	Post-treatment staging in rectal cancer	Mean: 68.5	Chemoradiotherapy
Li et al	2020	[ ^18^ F]FDG PET/MRI	Germany	Pro	PB	Pathology and follow-up imaging	34	Initial staging and Post-treatment staging in rectal cancer	Mean: 58	Chemotherapy/radiotherapy
Plodeck et al	2018	[ ^18^ F]FDG PET/MRI	Germany	Retro	PB	Pathology and follow-up imaging	44	Post-treatment staging in colorectal cancer	Mean: 60	Chemotherapy/radiotherapy/surgery
Kang et al	2016	[ ^18^ F]FDG PET/MRI	Korea	Retro	PB	Pathology and follow-up imaging	12	Initial staging and Post-treatment staging in Colorectal cancer	Mean: 60.2	NA
Brendle et al	2016	[ ^18^ F]FDGPET/MRI	Germany	Retro	LB	Follow-up imaging	15	Initial staging in colorectal cancer	Mean (range): 45 (10–62)	Surgery, chemotherapy, radiation
Lee et al	2015	[ ^18^ F]FDGPET/MRI	Korea	Retro	LB	Pathology	20	Initial staging in colorectal cancer	Mean: 58.3	NA

Abbreviations: LB, lesion-based; NA, not available; PB, person-based; Pro, prospective; Retro, retrospective.

**Table 2:** Technical aspects of included studies for [ ^18^ F]FDG PET/CT and [ ^18^ F]FDG PET/MRI

Author	Year	Types of imaging tests	Scanner Modality for PET	Radiotracer dose	TP	FP	FN	TN
Engel et al	2024	[ ^18^ F]FDG PET/CT	NA	NA	79	10	17	371
Nasr et al	2023	[ ^18^ F]FDG PET/CT	Philips Medical Systems with 16-slice CT	185 − 555 MBq	43	4	11	21
Gauci et al	2023	[ ^18^ F]FDG PET/CT	NA	NA	9	3	8	14
Gunduz et al	2023	[ ^18^ F]FDG PET/CT [ ^18^ F]FDG PET/MRI	Discovery 710, GE Health Japan (CT) SIGNA PET/MR, GE Healthcare (MRI)	296–370 MBq	31	1	10	36
Xu et al	2023	[ ^18^ F]FDG PET/CT	Biograph mCT, Siemens Healthcare	5.55 MBq/kg	33	28	19	52
Yukimoto et al	2022	[ ^18^ F]FDG PET/CT	Discovery 710, GE Health Japan	4.8 MBq/kg	129	177	55	148
Yukimoto et al	2021	[ ^18^ F]FDG PET/CT	LightSpeed VCT, GE Healthcare	370 MBq	14	10	3	141
Bae et al	2018	[ ^18^ F]FDG PET/CT	Discovery STE 16, GE Healthcare, Milwaukee, WI, USA and Biograph mCT 64, Siemens Healthcare, Knoxville, TN, USA	4.0 MBq/kg and 7.0 MBq/kg	51	28	16	81
Chen et al	2018	[ ^18^ F]FDG PET/CT	Biograph mCT, Siemens Medical Systems	3.7 MBq/kg	23	26	4	37
Atici et al	2016	[ ^18^ F]FDG PET/CT	Biograph mCT 64, Siemens Healthcare, Erlangen, Germany	296 − 703 MBq	13	0	16	25
Paspulati et al	2015	[ ^18^ F]FDG PET/CT	Gemini TF PET/CT scanner(CT)	352–525 MBq	5	2	0	5
Kwak et al	2012	[ ^18^ F]FDG PET/CT	Discovery PET/CT, GE Healthcare	7.4 MBq/kg	162	91	83	137
Kim et al	2011	[ ^18^ F]FDG PET/CT	Biograph Sensation 16TM and TruePoint 40, Siemens Medical Systems, Malvern, PA or Discovery STE 8, GE Healthcare, Piscataway, NJ, USA	370 MBq	26	13	23	144
Ono et al	2009	[ ^18^ F]FDG PET/CT	Advance Nxi PET Scanner, GE Healthcare, USA	3.7 MBq/kg	3	0	13	7
Akiyoshi et al	2008	[ ^18^ F]FDG PET/CT	ECAT Accel, Siemens, Malvern, Pennsylvania	200–350 MBq	15	1	20	20
Tsunoda et al	2008	[ ^18^ F]FDG PET/CT	Discovery LS8, GE Healthcare, Milwaukee, WI, USA	370 MBq	26	12	23	115
Gunduz et al	2023	[ ^18^ F]FDG PET/MRI	SIGNA PET/MR, GE Healthcare	296–370 MBq	41	0	0	36
Seto et al	2022	[ ^18^ F]FDG PET/MRI	SIGNA PET/MR, GE Healthcare	200 MBq	8	0	1	7
Catalano et al	2021	[ ^18^ F]FDG PET/MRI	Biograph mMR, Siemens Healthcare, Erlangen, Germany	4.44 MBq/kg	44	2	4	12
Crimì et al	2020	[ ^18^ F]FDG PET/MRI	Biograph mMR, Siemens	3 MBq/kg	10	2	1	23
Li et al	2020	[ ^18^ F]FDG PET/MRI	Biograph mMR, Siemens Healthcare, Germany	266.6 ± 58.8 MBq	7	1	4	11
Plodeck et al	2018	[ ^18^ F]FDG PET/MRI	Integrated whole-body PET/MRI, Siemens Healthcare	241–350 MBq	29	1	2	17
Kang et al	2016	[ ^18^ F]FDG PET/MRI	Integrated whole-body PET/MRI, Siemens Healthcare	5.18 MBq/kg	4	4	3	1
Brendle et al	2016	[ ^18^ F]FDG PET/MRI	Biograph mMR, Siemens Healthcare, Erlangen, Germany	337 ± 59 MBq	12	2	8	33
Lee et al	2015	[ ^18^ F]FDG PET/MRI	Biograph mMR, Siemens Healthcare, Germany	330 ± 51.8 MBq	10	1	1	8
Paspulati et al	2015	[ ^18^ F]FDG PET/MRI	Ingenuity TF PET/MRI (MRI)	352–525 MBq	6	1	0	5

Abbreviations: FN, false negative; FP, false positive; Mbq, megabecquerel; NA, not available; TN, true negative; TP, true positive.

**Fig. 2:**
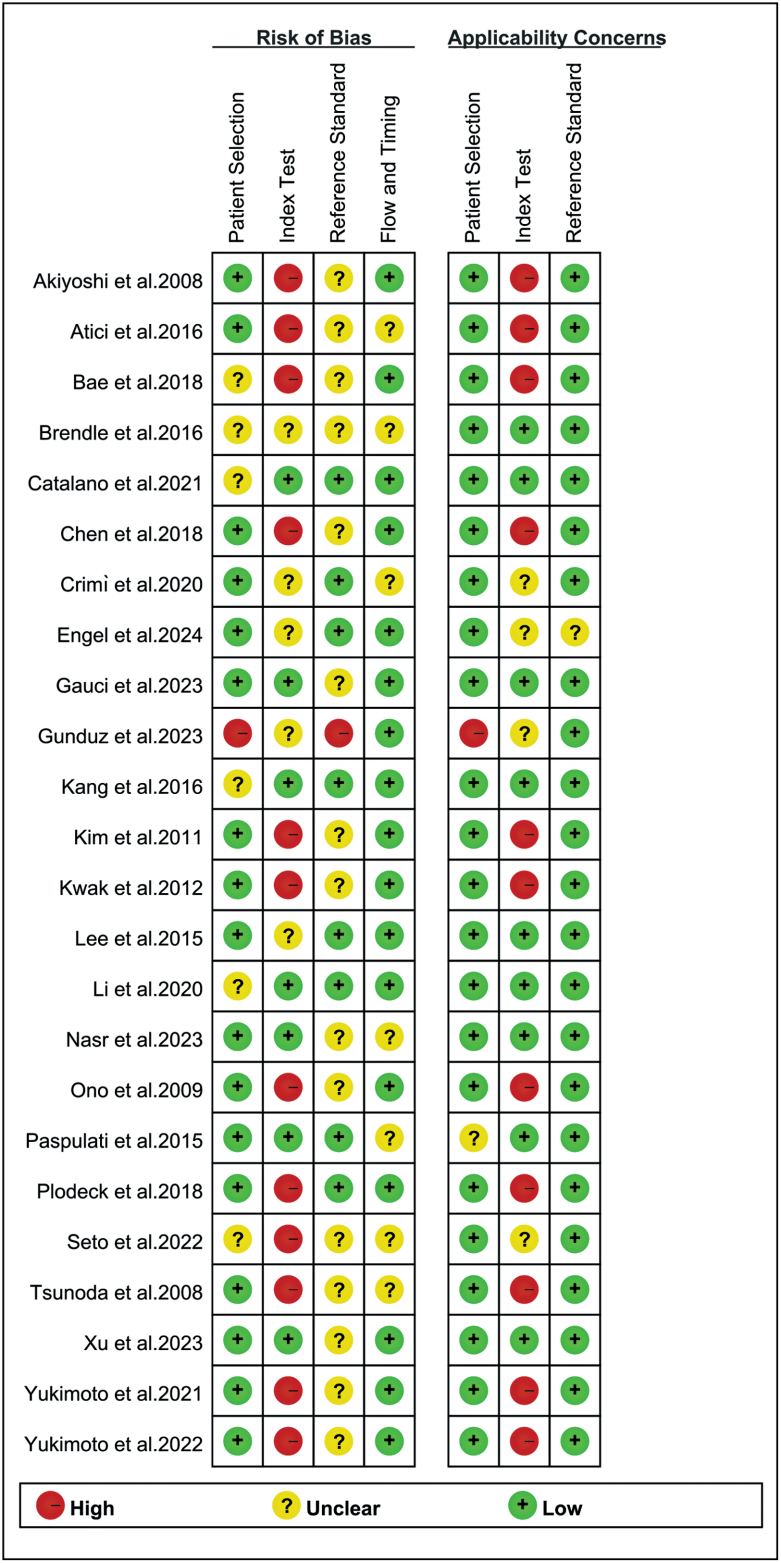
Risk of bias and applicability concerns of the included studies using the Quality Assessment of Diagnostic Performance Studies QUADAS-2 tool.

The risk of bias for each study was evaluated using the QUADAS-2 tool, as illustrated in Fig. 2 . In this assessment, 12 studies were classified as “high risk” due to the lack of predetermined cut-off values for the index test. 25 27 28 29 30 31 32 33 34 35 36 46 Additionally, 12 studies were rated as “high risk” for clinical applicability, attributed to inconsistent operational procedures and interpretations of the diagnostic tool. Despite these issues, the overall quality of the included studies did not raise significant concerns based on the comprehensive quality assessment.

### Sensitivity Comparison of [ ^18^ F]FDG PET/CT and [ ^18^ F]FDG PET/MRI for Detecting Lymph Node Metastases in CRC

**Fig. 3:**
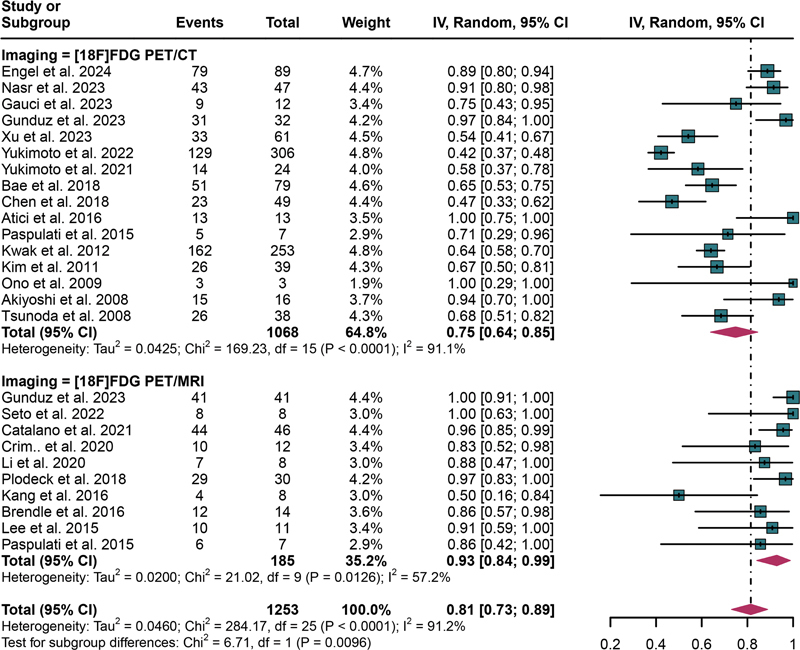
Forest plot showing the pooled sensitivities of [ ^18^ F]FDG PET/CT and [ ^18^ F]FDG PET/MRI in lymph metastasis of CRC patients. The plot displays individual study estimates (squares) with corresponding 95% confidence intervals (horizontal lines) and the pooled sensitivity estimate (diamond) for both modalities. The size of the squares represents the relative weight of each study in the meta-analysis.

The aggregated sensitivity of [ ^18^ F]FDG PET/CT for detecting lymph node metastases in CRC was 0.75 (95% CI: 0.64–0.85). Conversely, [ ^18^ F]FDG PET/MRI exhibited a higher pooled sensitivity of 0.93 (95% CI: 0.84–0.99; Fig. 3 ). Statistical analysis revealed a significant difference in sensitivity between [ ^18^ F]FDG PET/CT and [ ^18^ F]FDG PET/MRI ( *p* = 0.0096; Fig. 3 ). The overall sensitivity of [ ^18^ F]FDG PET/CT and [ ^18^ F]FDG PET/MRI demonstrated *I* ^2^ values of 91.1 and 91.2%, respectively, reflecting different degrees of heterogeneity. For PET/CT, meta-regression analysis indicated that the region (Asia vs. non-Asia, *p <* 0.01), the design of research implementation (retrospective vs. prospective, *p* < 0.01) and the location of the tumor (superior abdomen vs. inferior abdomen, *p* < 0.0001) might be the potential sources of this heterogeneity ( Table 3 ). Similarly, for PET/MRI, meta-regression analysis indicated that the location of the tumor (superior abdomen vs. inferior abdomen, *p* < 0.01) and the number of patients included (> 50 vs. ≤ 50, *p* = 0.02) could be the potential sources of this heterogeneity ( Table 4 ). The leave-one-out sensitivity analysis showed that removing the studies by Kang et al and Akkus Gunduz et al reduced the *I ^2^* to 38.8% and 31.0%, respectively, implying that these studies made a substantial contribution to the heterogeneity ( Supplementary Figs. S1 and S2 [available in the online version only]). 40 44

**Table 3:** Subgroup analysis and meta-regression analysis of [ ^18^ F]FDG PET/CT

Covariate	Studies, *n*	Sensitivity (95% CI)	*p* -Value	Specificity (95% CI)	*p* -Value
Number of patients included			0.44		0.69
>50	13	0.76 (0.63–0.87)		0.76 (0.65–0.86)	
≤50	3	0.70 (0.56–0.82)		0.82 (0.63–0.96)	
Region			<0.01		0.54
Asia	12	0.72 (0.58–0.83)		0.75 (0.65–0.85)	
Non-Asia	4	0.90(0.84-0.94)		0.84 (0.60–0.99)	
Study design			<0.01		0.14
Retrospective	13	0.70 (0.59–0.80)		0.79 (0.68–0.88)	
Prospective	3	0.96 (0.79–1.00)		1.00 (0.85–1.00)	
Tumor location			<0.0001		0.67
Superior abdomen	1	0.97 (0.84–1.00)		0.77 (0.68–0.85)	
Inferior abdomen	6	0.65 (0.60–0.69)		0.81 (0.68–0.92)	
Both	9	0.78 (0.60–0.93)		0.74 (0.58–0.87)	

**Table 4:** Subgroup analysis and meta-regression analysis of [ ^18^ F]FDG PET/MRI

Covariate	Studies, *n*	Sensitivity (95% CI)	*p* -Value	Specificity (95% CI)	*p* -Value
Number of patients included			0.02		0.61
>50	8	0.89 (0.79–0.97)		0.87 (0.77–0.94)	
≤50	2	0.99 (0.92–1.00)		0.93 (0.54–1.00)	
Region			0.89		0.93
East Asia	4	0.93 (0.66–1.00)		0.86 (0.50–1.00)	
Non-East Asia	6	0.94 (0.87–0.98)		0.86 (0.77–0.94)	
Study design			0.23		0.63
Retrospective	7	0.94 (0.84–1.00)		0.86 (0.70–0.97)	
Prospective	3	0.85 (0.68–0.98)		0.92 (0.72–1.00)	
Tumor location			<0.01		<0.01
Superior abdomen	1	1.00 (0.91–1.00)		1.00 (0.90–1.00)	
Inferior abdomen	5	0.96 (0.90–1.00)		0.86 (0.75–0.95)	
Both	4	0.81 (0.64–0.94)		0.82 (0.56–0.99)	

### Specificity Comparison of [ ^18^ F]FDG PET/CT and [ ^18^ F]FDG PET/MRI for Detecting Lymph Node Metastases in CRC

**Fig. 4:**
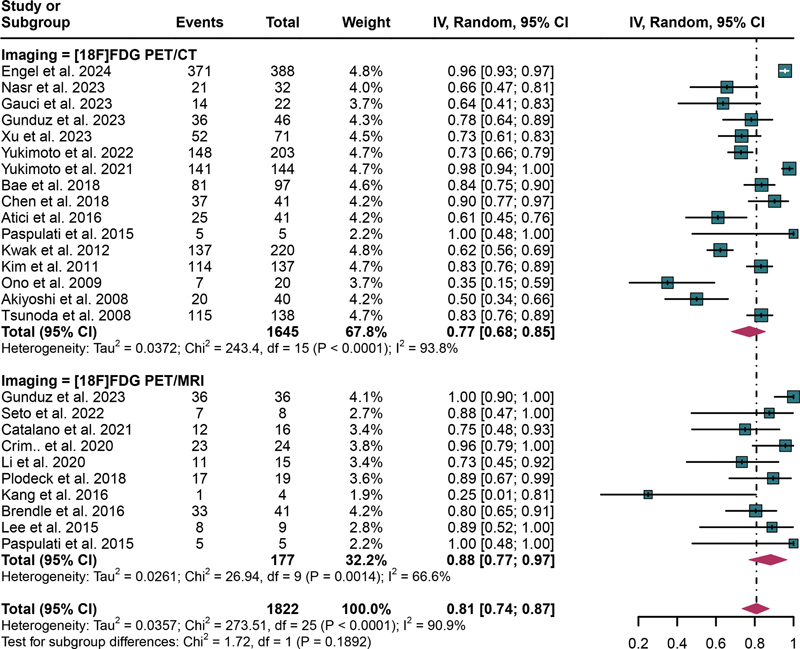
Forest plot showing the pooled specificities of [ ^18^ F]FDG PET/CT and [ ^18^ F]FDG PET/MRI in lymph metastasis of CRC patients. The plot displays individual study estimates (squares) with corresponding 95% confidence intervals (horizontal lines) and the pooled sensitivity estimate (diamond) for both modalities. The size of the squares represents the relative weight of each study in the meta-analysis.

The aggregated specificity of [ ^18^ F]FDG PET/CT for detecting lymph node metastases in CRC was 0.77 (95% CI: 0.68–0.85). Conversely, [ ^18^ F]FDG PET/MRI exhibited a higher pooled specificity of 0.88 (95% CI: 0.77–0.97, Fig. 4 ). The statistical analysis indicated no significant difference in specificity between [ ^18^ F]FDG PET/CT and [ ^18^ F]FDG PET/MRI ( *p* = 0.1892; Fig. 4 ). The overall sensitivity of [ ^18^ F]FDG PET/CT and [ ^18^ F]FDG PET/MRI exhibited *I ^2^* values of 93.8 and 66.6%, respectively, reflecting different degrees of heterogeneity. For PET/CT, meta-regression analysis failed to identify a potential source of heterogeneity. On the contrary, for PET/MRI, meta-regression analysis indicated that the location of the tumor (superior abdomen vs. inferior abdomen, *p* < 0.01; Tables 3 and 4 ) could be a contributing factor. The leave-one-out sensitivity analysis revealed that excluding the study by Akkus Gunduz et al decreased the *I* ^2^ to 37.1%, indicating that this study might be the source of heterogeneity ( Supplementary Figs. S3 and S4 [available in the online version only]).

### SROC Curve Results

**Fig. 5:**
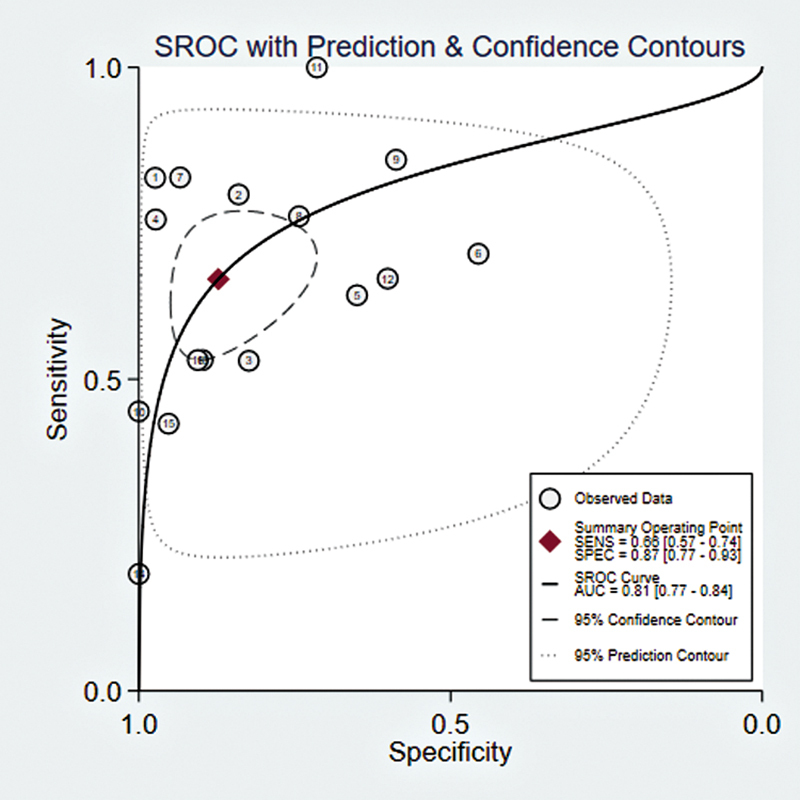
The forest plot of SROC curves of [ ^18^ F]FDG PET/CT for lymph metastases in patients with CRC.

**Fig. 6:**
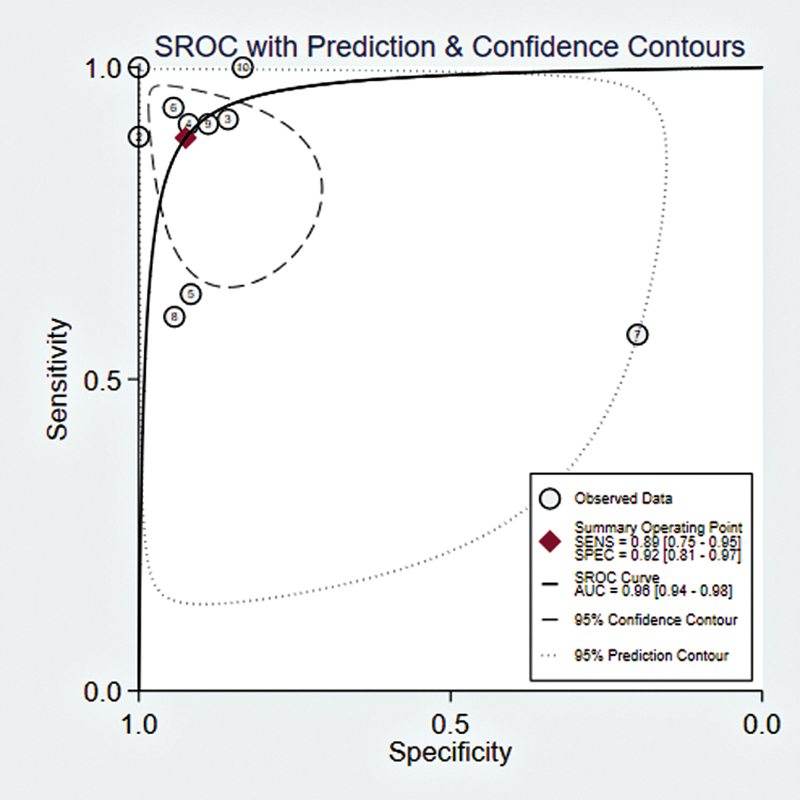
The forest plot of SROC curves of [ ^18^ F]FDG PET/MRI for lymph metastases in patients with CRC.

The forest plot of SROC curves results showed that the sensitivity and specificity of PET/CT diagnosis were 0.66 (95% CI: 0.57–0.74) and 0.87 (95% CI: 0.77–0.93), respectively, with an AUC of 0.81 (95% CI: 0.77–0.84). For PET/MRI diagnosis, the sensitivity and specificity were 0.89 (95% CI: 0.75–0.95) and 0.92 (95% CI: 0.81–0.97), respectively, with an AUC of 0.96 (95% CI: 0.94–0.98). The results indicated that PET/MRI diagnosis may be slightly superior to PET/CT ( Figs. 5 and 6 ).

### Publication Bias

The assessment of publication bias using funnel plot asymmetry revealed no significant bias across any of the outcomes, as indicated by Egger's test (all *p* > 0.05; Supplementary Figs. S5–S8 [available in the online version only]).

## Discussion

The diagnostic effectiveness of [ ^18^ F]FDG PET/CT and [ ^18^ F]FDG PET/MRI in identifying lymph node metastases in CRC remains a topic of ongoing debate and uncertainty within the medical community. Existing guidelines from the American Society of Clinical Oncology (ASCO) offer invaluable recommendations on imaging modalities for detecting lymph node metastases in CRC. 47 48 49 50 ASCO guidelines underscore PET/CT's utility in disease recurrence detection and exclusion of distant metastases, particularly in cases where conventional imaging yields inconclusive results. 47 48 While acknowledging PET/CT's established role, ASCO also recognizes the emerging potential of PET/MRI, especially in enhancing the staging of rectal cancer. 51 Recent research suggests that [ ^18^ F]FDG PET/MRI may offer advantages over PET/CT, notably in detecting small metastatic lesions and providing superior soft tissue contrast. 50 Our findings support this trend, revealing that [ ^18^ F]FDG PET/MRI had a higher diagnostic accuracy for detecting lymph node metastases than PET/CT with a higher sensitivity (0.75 vs. 0.93) and a higher AUC value (0.96 vs. 0.81).

To further clarify the diagnostic performance of [ ^18^ F]FDG PET/CT and [ ^18^ F]FDG PET/MRI in CRC lymph node metastases, we conducted a meta-regression analysis focusing on the abdomen. The sensitivity of PET/CT, calculated from six studies in the lower abdomen, was found to be 0.65 (0.60–0.69). This result was notably lower than the overall sensitivity of 0.75 (95% CI: 0.64–0.85) derived from a larger pool of 16 studies. This decline in performance could be mainly attributed to the physiological characteristics of the lower abdominal region. In the lower abdomen, the radiotracer used in PET/CT is excreted through the bladder. This physiological process leads to high-intensity signals in the bladder area, which can mask or interfere with the detection of lymph node metastases. 52 As a result, the accuracy of PET/CT in the lower abdomen is compromised, making it less effective compared with its performance in other regions. In contrast, PET/CT shows better applicability in the upper abdominal area, where the influence of such physiological factors is relatively less significant. Conversely, PET/MRI demonstrated a more favorable performance in the lower abdomen. Data from five out of ten relevant studies indicated a sensitivity of 0.96 (0.90–1.00), which is higher than its overall sensitivity of 0.93 (95% CI: 0.84–0.99). Pelvic MRI has a natural advantage in identifying nodal metastases. The high-resolution soft-tissue imaging provided by MRI allows for a more detailed visualization of lymph nodes, enabling the detection of even small-sized metastases. The multiplanar imaging capabilities of MRI also contribute to a more comprehensive assessment of the lymphatic system in the lower abdomen. 53 These findings have significant clinical implications. In the diagnosis of CRC lymph node metastases, the choice between [ ^18^ F]FDG PET/CT and [ ^18^ F]FDG PET/MRI should be carefully considered based on the location of the suspected metastases. For patients with suspected metastases in the upper abdomen, [ ^18^ F]FDG PET/CT can be a reliable imaging modality due to its relatively better performance in this region. However, when dealing with suspected metastases in the lower abdomen, [ ^18^ F]FDG PET/MRI should be given preference because of its superior sensitivity and accuracy.

In comparing our findings with prior studies, we note that our meta-analysis offers a more current and comprehensive analysis than earlier syntheses. For instance, while Dahmarde et al 54 included 13 studies exclusively evaluating [ ^18^ F]FDG PET/CT for identifying lymph node metastases in CRC, reporting a sensitivity and specificity of 0.65 and 0.75, respectively, our analysis incorporates additional studies published after 2020, ensuring an up-to-date synthesis of the latest research. Meanwhile, by comparing both PET/CT and PET/MRI modalities, our study offers a broader perspective, providing robust guidance for clinical practice by elucidating the relative strengths and weaknesses of each imaging technique. Rooney et al 55 conducted a meta-analysis across six studies on [ ^18^ F]FDG PET/CT, reporting a pooled sensitivity of 0.54 (95% CI: 0.47–0.70) and specificity of 0.95 (95% CI: 0.86–0.98). For [ ^18^ F]FDG PET/MRI, they reported a pooled sensitivity of 0.72 (95% CI: 0.51–0.87) and specificity of 0.90 (95% CI: 0.78–0.96), including comparisons with conventional CT and MRI for detecting lateral lymph node metastases in rectal cancer. However, due to the limited number of studies, further statistical analysis was constrained. In contrast, our meta-analysis, encompassing a greater number of included studies and a more recent timeframe, enhances its clinical applicability by providing improved timeliness and a comprehensive comparison of PET/CT and PET/MRI diagnostic performance in detecting lymph node metastases in CRC. This expanded scope enhances the relevance of our findings for guiding clinical decisions and advancing the field of imaging in CRC management. Furthermore, our study also conducted subgroup analyses to explore potential sources of heterogeneity among studies, such as differences in patient characteristics and imaging techniques. This subgroup analysis helps identify factors that may affect the performance of the two modalities and provides a more comprehensive understanding of the results.

While our meta-analysis suggests that [ ^18^ F]FDG PET/MRI offers higher sensitivity than [ ^18^ F]FDG PET/CT, it is critical to emphasize that PET/MRI remains significantly less widespread globally, particularly as growing interest in total-body PET/CT systems has further limited its clinical adoption. [ ^18^ F]FDG PET/MRI combines metabolic imaging from PET with the superior soft tissue contrast of MRI, which is particularly beneficial for detecting and characterizing soft tissue abnormalities. This provides clearer differentiation of tumor tissue from surrounding structures without the use of ionizing radiation, making [ ^18^ F]FDG PET/MRI an attractive option. 56 Interestingly, the detection rates of lymph nodes by both imaging modalities may also correlate with the biological characteristics of the tumor, particularly with tumor grade and aggressiveness. 57 For example, in high-grade tumors or those with more aggressive biological features, PET/MRI may demonstrate a higher sensitivity in identifying metastatic lymph nodes due to the improved soft tissue contrast provided by MRI. 58 On the contrary, [ ^18^ F]FDG PET/CT, which relies on the standard SUV (standardized uptake value) measurements, may exhibit limitations in distinguishing lymph node involvement in tumors with lower metabolic activity. These differences in sensitivity can be particularly pronounced in tumors with a heterogeneous or low FDG uptake profile, where the combination of MRI's tissue-specific contrast and PET's metabolic imaging may allow for a more accurate assessment of lymph node involvement. 59 However, its clinical applicability is constrained by contraindications such as incompatible metallic implants, claustrophobia, and renal dysfunction (when gadolinium contrast is needed), necessitating [ ^18^ F]FDG PET/CT for certain populations. In contrast, [ ^18^ F]FDG PET/CT's cost-effectiveness, faster scanning times, and global accessibility have solidified its role as a first-line modality. 60 Recent technological advancements, such as digital PET/CT scanners and advanced reconstruction algorithms, can cause variability in SUV and, therefore, detection rates. For instance, a millimetric lymph node may demonstrate higher radiotracer uptake when assessed with a dedicated reconstruction algorithm on a digital scanner, compared with the same lymph node evaluated on a PET/CT scanner from a decade ago. 61 Despite these innovations, PET/CT's reliance on ionizing radiation raises concerns for younger patients and those requiring repeated scans, as cumulative exposure may increase malignancy risks. 62 This exposure can increase the risk of radiation-induced malignancies, making PET/MRI a safer long-term option for these patient groups. Thus, while [ ^18^ F]PET/CT remains the pragmatic choice for routine clinical use, [ ^18^ F]PET/MRI's enhanced sensitivity and safety profile position it as a valuable alternative for specific scenarios.

When interpreting the results of our meta-analysis, several limitations need to be acknowledged. First, variability among the included studies may have affected the combined sensitivity and specificity estimates for [ ^18^ F]FDG PET/CT and [ ^18^ F]FDG PET/MRI. To explore the sources of this variability, we conducted meta-regression and sensitivity analyses. Leave-one-out sensitivity analysis identified certain studies, such as Kang et al and Akkus Gunduz et al, 40 44 as potential sources of heterogeneity, evidenced by the reduction in *I* ^2^ values after their exclusion. Additionally, the predominance of retrospective studies in our analysis introduces the risk of inherent bias. Furthermore, the absence of head-to-head comparison studies limits the direct comparison between PET/MRI and PET/CT. Therefore, well-designed prospective head-to-head studies are necessary to validate our findings and provide deeper insights into the diagnostic performance of these imaging modalities in the staging and management of CRC.

## Conclusion

Our meta-analysis suggests that [ ^18^ F]FDG PET/MRI has a higher sensitivity and comparable specificity to [ ^18^ F]FDG PET/CT for detecting lymph node metastases in patients of CRC. Nevertheless, the absence of direct comparative studies in this analysis underscores the necessity for future large-scale prospective research to validate these findings.

## References

[1] Keum N Giovannucci E Global burden of colorectal cancer: emerging trends, risk factors and prevention strategies Nat Rev Gastroenterol Hepatol 2019 16 12 713 732 31455888 10.1038/s41575-019-0189-8

[2] Roshandel G Ghasemi-Kebria F Malekzadeh R Colorectal cancer: epidemiology, risk factors, and prevention Cancers (Basel) 2024 16 08 1530 38672612 10.3390/cancers16081530 PMC11049480

[3] Dekker E Tanis P J Vleugels J LA Kasi P M Wallace M B Colorectal cancer Lancet 2019 394(10207):1467 1480 31631858 10.1016/S0140-6736(19)32319-0

[4] Sueda T Tei M Yasuyama A Impact of regional lymph node metastasis on pulmonary metastasis as the first recurrence site Updates Surg 2023 75 07 1843 1855 37615847 10.1007/s13304-023-01633-1

[5] Orlandi E Giuffrida M Trubini S Unraveling the Interplay of KRAS, NRAS, BRAF, and micro-satellite instability in non-metastatic colon cancer: a systematic review Diagnostics (Basel) 2024 14 10 1001 38786299 10.3390/diagnostics14101001 PMC11120454

[6] Simon K Colorectal cancer development and advances in screening Clin Interv Aging 2016 11 967 976 27486317 10.2147/CIA.S109285 PMC4958365

[7] Mahmoud N N Colorectal cancer: preoperative evaluation and staging Surg Oncol Clin N Am 2022 31 02 127 141 35351269 10.1016/j.soc.2021.12.001

[8] Bando K Nishimoto R Matsuhiro M Metastatic lymph node analysis of colorectal cancer using quadruple-phase CT imagesIn: Medical Imaging;Bellingham, WA, USA SPIE – The International Society for Optics and Photonics 2019

[9] Jung W Park K R Lee K J Value of imaging study in predicting pelvic lymph node metastases of uterine cervical cancer Radiat Oncol J 2017 35 04 340 348 29232805 10.3857/roj.2017.00206 PMC5769886

[10] Jain S Maque J Galoosian A Osuna-Garcia A May F P Optimal strategies for colorectal cancer screening Curr Treat Options Oncol 2022 23 04 474 493 35316477 10.1007/s11864-022-00962-4 PMC8989803

[11] Miyazaki K Wada Y Okuno K An exosome-based liquid biopsy signature for pre-operative identification of lymph node metastasis in patients with pathological high-risk T1 colorectal cancer Mol Cancer 2023 22 01 2 36609320 10.1186/s12943-022-01685-8 PMC9817247

[12] García-Figueiras R Baleato-González S Padhani A R Advanced imaging of colorectal cancer: from anatomy to molecular imaging Insights Imaging 2016 7 03 285 309 27136925 10.1007/s13244-016-0465-x PMC4877344

[13] Mainenti P P Stanzione A Guarino S Colorectal cancer: parametric evaluation of morphological, functional and molecular tomographic imaging World J Gastroenterol 2019 25 35 5233 5256 31558870 10.3748/wjg.v25.i35.5233 PMC6761241

[14] Kijima S Sasaki T Nagata K Utano K Lefor A T Sugimoto H Preoperative evaluation of colorectal cancer using CT colonography, MRI, and PET/CT World J Gastroenterol 2014 20 45 16964 16975 25493009 10.3748/wjg.v20.i45.16964 PMC4258565

[15] Kumamoto T Shindoh J Mita H Optimal diagnostic method using multidetector-row computed tomography for predicting lymph node metastasis in colorectal cancer World J Surg Oncol 2019 17 01 39 30795767 10.1186/s12957-019-1583-y PMC6387477

[16] Crimì F Valeggia S Baffoni L [ ^18^ F]FDG PET/MRI in rectal cancer Ann Nucl Med 2021 35 03 281 290 33517562 10.1007/s12149-021-01580-0 PMC7902586

[17] Scarsbrook A F Barrington S F PET-CT in the UK: current status and future directions Clin Radiol 2016 71 07 673 690 27044903 10.1016/j.crad.2016.02.023

[18] van Sluis J Borra R Tsoumpas C Extending the clinical capabilities of short- and long-lived positron-emitting radionuclides through high sensitivity PET/CT Cancer Imaging 2022 22 01 69 36527149 10.1186/s40644-022-00507-w PMC9755796

[19] Mirshahvalad S A Hinzpeter R Kohan A Diagnostic performance of [ ^18^ F]-FDG PET/MR in evaluating colorectal cancer: a systematic review and meta-analysis Eur J Nucl Med Mol Imaging 2022 49 12 4205 4217 35705874 10.1007/s00259-022-05871-0

[20] Baghdadi A Mirpour S Ghadimi M Imaging of colorectal liver metastasis J Gastrointest Surg 2022 26 01 245 257 34664191 10.1007/s11605-021-05164-1

[21] Buchbender C Heusner T A Lauenstein T C Bockisch A Antoch G Oncologic PET/MRI, part 2: bone tumors, soft-tissue tumors, melanoma, and lymphoma J Nucl Med 2012 53 08 1244 1252 22782313 10.2967/jnumed.112.109306

[22] Salameh J P Bossuyt P M McGrath T A Preferred reporting items for systematic review and meta-analysis of diagnostic test accuracy studies (PRISMA-DTA): explanation, elaboration, and checklist BMJ 2020 370 m2632 32816740 10.1136/bmj.m2632

[23] Nasr I M Maksoud B A Rezk M A Badawy A Almorsy W A Ali I M 18F-FDG PET/CT in therapy response assessment: oligometastatic colorectal cancerEgypt J Radiol Nucl Med 2023;54(1)

[24] Gauci C M Kim T J Gao Y Perera D S Accuracy of pre-operative 18-fluoride fluorodeoxyglucose positron emission tomography (FDG-PET) in predicting lymph node involvement in colon cancer ANZ J Surg 2023 93 11 2675 2679 37530228 10.1111/ans.18637

[25] Xu L Huang G Wang Y Huang G Liu J Chen R 2-[ ^18^ F]FDG PET-based quantification of lymph node metabolic heterogeneity for predicting lymph node metastasis in patients with colorectal cancer Eur J Nucl Med Mol Imaging 2024 51 06 1729 1740 38150017 10.1007/s00259-023-06578-6

[26] Yukimoto R Uemura M Tsuboyama T Efficacy of PET/CT in diagnosis of regional lymph node metastases in patients with colorectal cancer: retrospective cohort study BJS Open 2022 6 04 zrac090 35950556 10.1093/bjsopen/zrac090 PMC9366635

[27] Yukimoto R Uemura M Tsuboyama T Efficacy of positron emission tomography in diagnosis of lateral lymph node metastases in patients with rectal cancer: a retrospective study BMC Cancer 2021 21 01 520 33962569 10.1186/s12885-021-08278-6 PMC8105987

[28] Chen R Wang Y Zhou X Huang G Liu J Preoperative PET/CT ^18^ F-FDG standardized uptake by lymph nodes as a significant prognostic factor in patients with colorectal cancer Contrast Media Mol Imaging 2018 2018 5.802109E6 10.1155/2018/5802109 PMC6236575 30515068

[29] Bae S U Won K S Song B-I Jeong W K Baek S K Kim H W Accuracy of F-18 FDG PET/CT with optimal cut-offs of maximum standardized uptake value according to size for diagnosis of regional lymph node metastasis in patients with rectal cancer Cancer Imaging 2018 18 01 32 30217167 10.1186/s40644-018-0165-5 PMC6137872

[30] Atici A E Cakir T Reyhan E Preoperative use of PET/CT in patients with colorectal and gastric cancer and its impact on treatment decision making Int Surg 2016 101(7–8):318 327

[31] Kwak J Y Kim J S Kim H J Ha H K Yu C S Kim J C Diagnostic value of FDG-PET/CT for lymph node metastasis of colorectal cancer World J Surg 2012 36 08 1898 1905 22526032 10.1007/s00268-012-1575-3

[32] Kim D J Kim J H Ryu Y H Jeon T J Yu J S Chung J J Nodal staging of rectal cancer: high-resolution pelvic MRI versus ^18^ F-FDGPET/CT J Comput Assist Tomogr 2011 35 05 531 534 21926843 10.1097/RCT.0b013e318225720f

[33] Ono K Ochiai R Yoshida T Comparison of diffusion-weighted MRI and 2-[fluorine-18]-fluoro-2-deoxy-D-glucose positron emission tomography (FDG-PET) for detecting primary colorectal cancer and regional lymph node metastases J Magn Reson Imaging 2009 29 02 336 340 19161185 10.1002/jmri.21638

[34] Akiyoshi T Oya M Fujimoto Y Comparison of preoperative whole-body positron emission tomography with MDCT in patients with primary colorectal cancer Colorectal Dis 2009 11 05 464 469 18637927 10.1111/j.1463-1318.2008.01643.x

[35] Tsunoda Y Ito M Fujii H Kuwano H Saito N Preoperative diagnosis of lymph node metastases of colorectal cancer by FDG-PET/CT Jpn J Clin Oncol 2008 38 05 347 353 18424814 10.1093/jjco/hyn032

[36] Seto S Tsujikawa T Sawai K Feasibility of [ ^18^ F]FDG PET/MRI with early-delayed and extended PET as one-stop imaging for staging and predicting metastasis in rectal cancer Oncology 2022 100 04 212 220 35086111 10.1159/000522205

[37] Catalano O A Lee S I Parente C Improving staging of rectal cancer in the pelvis: the role of PET/MRI Eur J Nucl Med Mol Imaging 2021 48 04 1235 1245 33034673 10.1007/s00259-020-05036-x

[38] Crimì F Spolverato G Lacognata C 18F-FDG PET/MRI for rectal cancer TNM restaging after preoperative chemoradiotherapy: initial experience Dis Colon Rectum 2020 63 03 310 318 31842163 10.1097/DCR.0000000000001568

[39] Li Y Mueller L I Neuhaus J P ^18^ F-FDG PET/MR versus MR alone in whole-body primary staging and restaging of patients with rectal cancer: What is the benefit of PET? J Clin Med 2020 9 10 3163 33003615 10.3390/jcm9103163 PMC7599654

[40] Kang B Lee J M Song Y S Added value of integrated whole-body PET/MRI for evaluation of colorectal cancer: comparison with contrast-enhanced MDCT AJR Am J Roentgenol 2016 206 01 W10 W20 26700358 10.2214/AJR.14.13818

[41] Brendle C Schwenzer N F Rempp H Assessment of metastatic colorectal cancer with hybrid imaging: comparison of reading performance using different combinations of anatomical and functional imaging techniques in PET/MRI and PET/CT in a short case series Eur J Nucl Med Mol Imaging 2016 43 01 123 132 26224536 10.1007/s00259-015-3137-z

[42] Lee S J Seo H J Kang K W Clinical performance of whole-body 18F-FDG PET/Dixon-VIBE, T1-weighted, and T2-weighted MRI protocol in colorectal cancer Clin Nucl Med 2015 40 08 e392 e398 26018689 10.1097/RLU.0000000000000812

[43] Engel R Kudura K Antwi K Diagnostic accuracy and treatment benefit of PET/CT in staging of colorectal cancer compared to conventional imaging Surg Oncol 2024 57 102151 39418774 10.1016/j.suronc.2024.102151

[44] Akkus Gunduz P Ozkan E Kuru Oz D Clinical value of fluorine-18-fluorodeoxyglucose PET/MRI for liver metastasis in colorectal cancer: a prospective study Nucl Med Commun 2023 44 02 150 160 36630219 10.1097/MNM.0000000000001651

[45] Paspulati R M Partovi S Herrmann K A Krishnamurthi S Delaney C P Nguyen N C Comparison of hybrid FDG PET/MRI compared with PET/CT in colorectal cancer staging and restaging: a pilot study Abdom Imaging 2015 40 06 1415 1425 26112492 10.1007/s00261-015-0474-0

[46] Plodeck V Rahbari N N Weitz J FDG-PET/MRI in patients with pelvic recurrence of rectal cancer: first clinical experiences Eur Radiol 2019 29 01 422 428 29980927 10.1007/s00330-018-5589-6

[47] Chiorean E G Nandakumar G Fadelu T Treatment of patients with late-stage colorectal cancer: ASCO resource-stratified guideline JCO Glob Oncol 2020 6 414 438 32150483 10.1200/JGO.19.00367 PMC7124947

[48] On Behalf of the Clinical Practice Guidelines Committee of the American Society of Colon and Rectal Surgeons You Y N Hardiman K M Bafford A The American Society of Colon and Rectal Surgeons Clinical Practice Guidelines for the management of rectal cancer Dis Colon Rectum 2020 63 09 1191 1222 33216491 10.1097/DCR.0000000000001762

[49] Benson A B Venook A P Al-Hawary M M Rectal cancer, version 2.2022, NCCN Clinical Practice Guidelines in Oncology J Natl Compr Canc Netw 2022 20 10 1139 1167 36240850 10.6004/jnccn.2022.0051

[50] Hope T A Kassam Z Loening A McNamara M M Paspulati R The use of PET/MRI for imaging rectal cancer Abdom Radiol (NY) 2019 44 11 3559 3568 31201431 10.1007/s00261-019-02089-x PMC7001508

[51] El-Shami K Oeffinger K C Erb N L American Cancer Society Colorectal Cancer Survivorship Care Guidelines CA Cancer J Clin 2015 65 06 428 455 26348643 10.3322/caac.21286 PMC5385892

[52] Wahl R L Why nearly all PET of abdominal and pelvic cancers will be performed as PET/CT J Nucl Med 2004 45 01 82S 95S 14736839

[53] Bruno F Arrigoni F Mariani S Advanced magnetic resonance imaging (MRI) of soft tissue tumors: techniques and applications Radiol Med 2019 124 04 243 252 30949892 10.1007/s11547-019-01035-7

[54] Dahmarde H Parooie F Salarzaei M Is ^18^ F-FDG PET/CT an accurate way to detect lymph node metastasis in colorectal cancer: a systematic review and meta-analysis Contrast Media Mol Imaging 2020 2020 5.439378E6 10.1155/2020/5439378 PMC7383332 32733174

[55] Rooney S Meyer J Afzal Z The role of preoperative imaging in the detection of lateral lymph node metastases in rectal cancer: a systematic review and diagnostic test meta-analysis Dis Colon Rectum 2022 65 12 1436 1446 36102825 10.1097/DCR.0000000000002537

[56] Jayaprakasam V S Ince S Suman G PET/MRI in colorectal and anal cancers: an update Abdom Radiol (NY) 2023 48 12 3558 3583 37062021 10.1007/s00261-023-03897-y

[57] Choi S H Moon W K Contrast-enhanced MR imaging of lymph nodes in cancer patients Korean J Radiol 2010 11 04 383 394 20592922 10.3348/kjr.2010.11.4.383 PMC2893309

[58] Margolis N E Moy L Sigmund E E Assessment of aggressiveness of breast cancer using simultaneous 18F-FDG-PET and DCE-MRI: preliminary observation Clin Nucl Med 2016 41 08 e355 e361 27187730 10.1097/RLU.0000000000001254 PMC4935605

[59] Gaeta C M Vercher-Conejero J L Sher A C Kohan A Rubbert C Avril N Recurrent and metastatic breast cancer PET, PET/CT, PET/MRI: FDG and new biomarkers Q J Nucl Med Mol Imaging 2013 57 04 352 366 24322792

[60] Martin O Schaarschmidt B M Kirchner J PET/MRI versus PET/CT for whole-body staging: results from a single-center observational study on 1,003 sequential examinations J Nucl Med 2020 61 08 1131 1136 31806777 10.2967/jnumed.119.233940

[61] De Ponti E Crivellaro C Morzenti S Clinical application of a high sensitivity BGO PET/CT scanner: effects of acquisition protocols and reconstruction parameters on lesions quantification Curr Radiopharm 2022 15 03 218 227 34994322 10.2174/1874471015666220107100200

[62] Oh J S Koea J B Radiation risks associated with serial imaging in colorectal cancer patients: should we worry? World J Gastroenterol 2014 20 01 100 109 24415862 10.3748/wjg.v20.i1.100 PMC3885998

