# Supplementary material for: Diagnostic Performance of [ 18 F]FDG PET/MRI and [ 18 F]FDG PET/CT in the Detection of Lymph Node Metastases in Colorectal Cancer: A Meta-analysis
Source: World J Nucl Med. 2025 Nov 12;24(4):279–92. doi: 10.1055/s-0045-1812491 (PMC12774531; doi:10.1055/s-0045-1812491)
Supplement: Supplementary file 1 — Supplementary Material [file 10-1055-s-0045-1812491-s2580007.pdf]

PRISMA 2020 Checklist

Section/Topic	Item no.	Checklist item	Reported on page number/line number	Reported on section/paragraph
Title				
Title	1	Identify the report as a systematic review	Page 1/line 1–3	Title
Abstract				
Abstract	2	See the ►**PRISMA 2020 for Abstracts checklist (Table 2)**	Page 1–2/line 26–50	Abstract
Introduction				
Rationale	3	Describe the rationale for the review in the context of existing knowledge	Page 2–3/line 55–86	Introduction
Objectives	4	Provide an explicit statement of the objective(s) or question(s) the review addresses	Page 3/line 87–90	Introduction
Methods				
Eligibility criteria	5	Specify the inclusion and exclusion criteria for the review and how studies were grouped for the syntheses	Page 3–4/line 104–110	Methods/Inclusion and Exclusion Criteria
Information sources	6	Specify all databases, registers, websites, organizations, reference lists, and other sources searched or consulted to identify studies. Specify the date when each source was last searched or consulted	Page 3/line 97–98	Methods/Search Strategy
Search strategy	7	Present the full search strategies for all databases, registers, and websites, including any filters and limits used	Page 3/line 94–101	Methods/Search Strategy
Selection process	8	Specify the methods used to decide whether a study met the inclusion criteria of the review, including how many reviewers screened each record and each report retrieved, whether they worked independently, and, if applicable, details of automation tools used in the process	Page 4/line 111–114	Methods/Inclusion and Exclusion Criteria
Data collection process	9	Specify the methods used to collect data from reports, including how many reviewers collected data from each report, whether they worked independently, any processes for obtaining or confirming data from study investigators, and, if applicable, details of automation tools used in the process	Page 4/line 124–129	Methods/Data Extraction
Data items	10a	List and define all outcomes for which data were sought. Specify whether all results that were compatible with each outcome domain in each study were sought (e.g., for all measures, time points, analyses), and if not, the methods used to decide which results to collect	Page 4/line 124–128	Methods/Data Extraction
	10b	List and define all other variables for which data were sought (e.g., participant and intervention characteristics, funding sources). Describe any assumptions made about any missing or unclear information	Page 4/line 124–128	Methods/Data Extraction

(*Continued*)

 © 2025. The Author(s).

(*Continued*)

Section/Topic	Item no.	Checklist item	Reported on page number/line number	Reported on section/ paragraph
Study risk of bias assessment	11	Specify the methods used to assess risk of bias in the included studies, including details of the tool(s) used, how many reviewers assessed each study, and whether they worked independently, and if applicable, details of automation tools used in the process	Page 4/line 140–142	Methods/Statistical Analysis
Effect measures	12	Specify for each outcome the effect measure(s) (e.g., risk ratio, mean difference) used in the synthesis or presentation of results	Page 4/line 136–139	Methods/Statistical Analysis
Synthesis methods	13a	Describe the processes used to decide which studies were eligible for each synthesis	Page 4/line 111–114	Methods/Inclusion and Exclusion Criteria
	13b	Describe any methods required to prepare the data for presentation or synthesis, such as handling of missing summary statistics or data conversions	Page 4/line 132–136	Methods/Statistical Analysis
	13c	Describe any methods used to tabulate or visually display results of individual studies and syntheses.	Page 4/line 124–128	Methods/Data Extraction
	13d	Describe any methods used to synthesize results and provide a rationale for the choice(s). If meta-analysis was performed, describe the model(s), method(s) to identify the presence and extent of statistical heterogeneity, and software package(s) used.	Page 4/line 132–142	Methods/Statistical Analysis
	13e	Describe any methods used to explore possible causes of heterogeneity among study results.	Page 4/line 138–139	Methods/Statistical Analysis
	13f	Describe any sensitivity analyses conducted to assess robustness of the synthesized results.	Page 4/line 140–141	Methods/Statistical Analysis
Reporting bias assessment	14	Describe any methods used to assess risk of bias due to missing results in a synthesis (arising from reporting biases).	Page 4/line 117–121	Methods/Quality Assessment
Certainty assessment	15	Describe any methods used to assess certainty (or confidence) in the body of evidence for an outcome.	Page 4/line 137–139	Methods/Statistical Analysis
Results				
Study selection	16a	Describe the results of the search and selection process, from the number of records identified in the search to the number of studies included in the review, ideally using a flow diagram	Page 4–5/line 146–151	Results/Study Selection and Data Extraction
	16b	Cite studies that met many but not all inclusion criteria (“near-misses”) and explain why they were excluded	Page 4–5/line 147–149	Results/Study Selection and Data Extraction
Study selection and data extraction				
Study characteristics	17	Cite each included study and present its characteristics	Page 5/line 154–163	Results/Study Description and Quality Assessment

(*Continued*)

Section/Topic	Item no.	Checklist item	Reported on page number/line number	Reported on section/ paragraph
Risk of bias in studies	18	Present assessments of risk of bias for each included study	Page 5/line 164–169	Results/Study Description and Quality Assessment
Results of individual studies	19	For all outcomes, present, for each study: (a) summary statistics for each group (where appropriate) and (b) an effect estimate and its precision (e.g., confidence/credible interval), ideally using structured tables or plots	Page 5–6/line 173–175,187–189	Results
Results of syntheses	20a	For each synthesis, briefly summarize the characteristics and risk of bias among contributing studies	Page 5/line 154–169	Results/Study Description and Quality Assessment
	20b	Present the results of all statistical syntheses conducted. If meta-analysis was done, present for each the summary estimate and its precision (e.g., confidence/credible interval) and measures of statistical heterogeneity. If comparing groups, describe the direction of the effect	Page 6/line 198–203	Results/SROC Curve Results
	20c	Present the results of all investigations of possible causes of heterogeneity among study results	Page 5–6/line 178–183, 192–195	Results
	20d	Present the results of all sensitivity analyses conducted to assess the robustness of the synthesized results	Page 5–6/line 178–183, 192–195	Results
Reporting biases	21	Present assessments of risk of bias due to missing results (arising from reporting biases) for each synthesis assessed	Page 6/line 206–207	Results/Publication Bias
Certainty of evidence	22	Present assessments of certainty (or confidence) in the body of evidence for each outcome assessed	Page 5–6/line 178–183, 192–195	Results
Discussion				
Discussion	23a	Provide a general interpretation of the results in the context of other evidence	Page 6/line 210–222	Discussion
	23b	Discuss any limitations of the evidence included in the review	Page 8/line 262–272	Discussion
	23c	Discuss any limitations of the review processes used	Page 8/line 262–272	Discussion
	23d	Discuss implications of the results for practice, policy, and future research	Page 7/line 242–261	Discussion
Other information				
Registration and protocol	24a	Provide registration information for the review, including the register name and registration number, or state that the review was not registered	Page 3/line 94–96	Methods/Search Strategy
	24b	Indicate where the review protocol can be accessed, or state that a protocol was not prepared.	Page 3/line 100–101	Methods/Search Strategy
	24c	Describe and explain any amendments to information provided at registration or in the protocol	Page 3/line 100–101	Methods/Search Strategy
Support	25	Describe sources of financial or non-financial support for the review, and the role of the funders or sponsors in the review	Page 8/line 281–284	Acknowledgments

(*Continued*)

 © 2025. The Author(s).

(*Continued*)

Section/Topic	Item no.	Checklist item	Reported on page number/line number	Reported on section/ paragraph
Competing interests	26	Declare any competing interests of review authors	Page 8/line 289–290	Footnote
Availability of data, code, and other materials	27	Report which of the following are publicly available and where they can be found: template data collection forms; data extracted from included studies; data used for all analyses; analytic code; any other materials used in the review	Page 8/line 294–299	Footnote

Table 2 PRISMA 2020 for Abstracts checklist

Section/Topic	Item no.	Checklist item	Reported on page number/line number	Reported on section/paragraph
**Title**				
Title	1	Identify the report as a systematic review.	Page 1/Line 1–3	Title
**Background**				
Objectives	2	Provide an explicit statement of the main objective(s) or question(s) the review addresses.	Page 1/line 30–32	Abstract/Background
**Methods**				
Eligibility criteria	3	Specify the inclusion and exclusion criteria for the review.	Page 3/line 103–110	Methods
Information sources	4	Specify the information sources (e.g., databases, registers) used to identify studies and the date when each was last searched.	Page 1/line 33–34	Abstract/Methods
Risk of bias	5	Specify the methods used to assess risk of bias in the included studies.	Page 1/line 37–38	Abstract/Methods
Synthesis of results	6	Specify the methods used to present and synthesize results	Page 1/line 38–39	Abstract/Methods
**Results**				
Included studies	7	Give the total number of included studies and participants and summarize relevant characteristics of studies	Page 1/line 40	Abstract/Results
Synthesis of results	8	Present results for main outcomes, preferably indicating the number of included studies and participants for each. If a meta-analysis was done, report the summary estimate and confidence/credible interval. If comparing groups, indicate the direction of the effect (i.e., which group is favored)	Page 1/line 40–46	Abstract/Results
**Discussion**				
Limitations of evidence	9	Provide a brief summary of the limitations of the evidence included in the review (e.g., study risk of bias, inconsistency, and imprecision)	Page 1/line 49–50	Abstract/Conclusions
Interpretation	10	Provide a general interpretation of the results and important implications	Page 1/line 47–49	Abstract/Conclusions

Table 2 (*Continued*)

Section/Topic	Item no.	Checklist item	Reported on page number/line number	Reported on section/paragraph
**Other**				
Funding	11	Specify the primary source of funding for the review.	Page 8/line 281–284	Acknowledgments
Registration	12	Provide the register name and registration number	Page 3/line 94–96	Methods

Supplementary Table S1 Search strategy in PubMed, Embase, and Web of Science

Database	Search strategy
PubMed	(“Positron Emission Tomography Computed Tomography”[Mesh] OR “PET-CT”[Title/Abstract] OR “PET-MRI”[Title/Abstract] OR “positron emission tomography magnetic resonance imaging”[Title/Abstract] OR “PET-MR”[Title/Abstract] OR “positron emission tomography magnetic resonance”[Title/Abstract]) AND (“Lymphatic Metastasis”[Mesh] OR “lymph node metastases”[Title/Abstract] OR “Lymph Node Metastasis”[Title/Abstract] OR “Lymph Node Metastases”[Title/Abstract] OR “metastatic lymph nodes”[Title/Abstract] OR “nodal metastases”[Title/Abstract] OR “LNM”[Title/Abstract]) AND (“Colorectal Neoplasms”[Mesh] OR “Colorectal Tumor”[Title/Abstract] OR “Colorectal Neoplasm”[Title/Abstract] OR “Colorectal Cancer”[Title/Abstract] OR “Rectal Neoplasms”[Mesh] OR “Rectal”[Title/Abstract] OR “Rectum”[Title/Abstract] OR “Colonic Neoplasms”[Mesh] OR “Colonic”[Title/Abstract] OR “Colon”[Title/Abstract])
Embase	(’PET-CT scanner’/exp OR ’positron emission tomography-computed tomography’/exp OR ’positron emission tomography/computed tomography’:ab,ti OR ’PET/CT’:ab,ti OR ’PET-MRI scanner’/exp OR ’PET-MRI’ OR ’positron emission tomography magnetic resonance imaging’:ab,ti OR ’PET-MR’:ab,ti OR ’positron emission tomography magnetic resonance’:ab,ti) AND (’lymph node metastasis’/exp OR ’lymph node metastases’:ab,ti OR ’Lymph Node Metastasis’:ab,ti OR ’Lymph Node Metastases’:ab,ti OR ’metastatic lymph nodes’:ab,ti OR ’nodal metastases’:ab,ti OR ’LNM’:ab,ti) AND (’colorectal tumor’/exp OR ’Colorectal Tumor’:ab,ti OR ’Colorectal Neoplasm’:ab,ti OR ’Colorectal Cancer’:ab,ti OR ’rectum tumor’/exp OR ’Rectal’:ab,ti OR “Rectum’:ab,ti OR ’colon tumor’/exp OR ’colon’:ab,ti OR ’colonic’:ab,ti)
Web of Science	((TS = (“Positron Emission Tomography Computed Tomography” OR “PET-CT” OR “PET-MRI” OR “positron emission tomography magnetic resonance imaging” OR “PET-MR” OR “positron emission tomography magnetic resonance”)) AND TS = (“Lymphatic Metastasis” OR “lymph node metastases” OR “Lymph Node Metastasis” OR “Lymph Node Metastases” OR “metastatic lymph nodes” OR “nodal metastases” OR “LNM”)) AND TS = (“Colorectal Neoplasms” OR “Colorectal Tumor” OR “Colorectal Neoplasm” OR “Colorectal Cancer” OR “Rectal Neoplasms” OR “Rectal” OR “Rectum” OR “Colonic Neoplasms” OR “Colonic” OR “Colon”)

 © 2025. The Author(s).

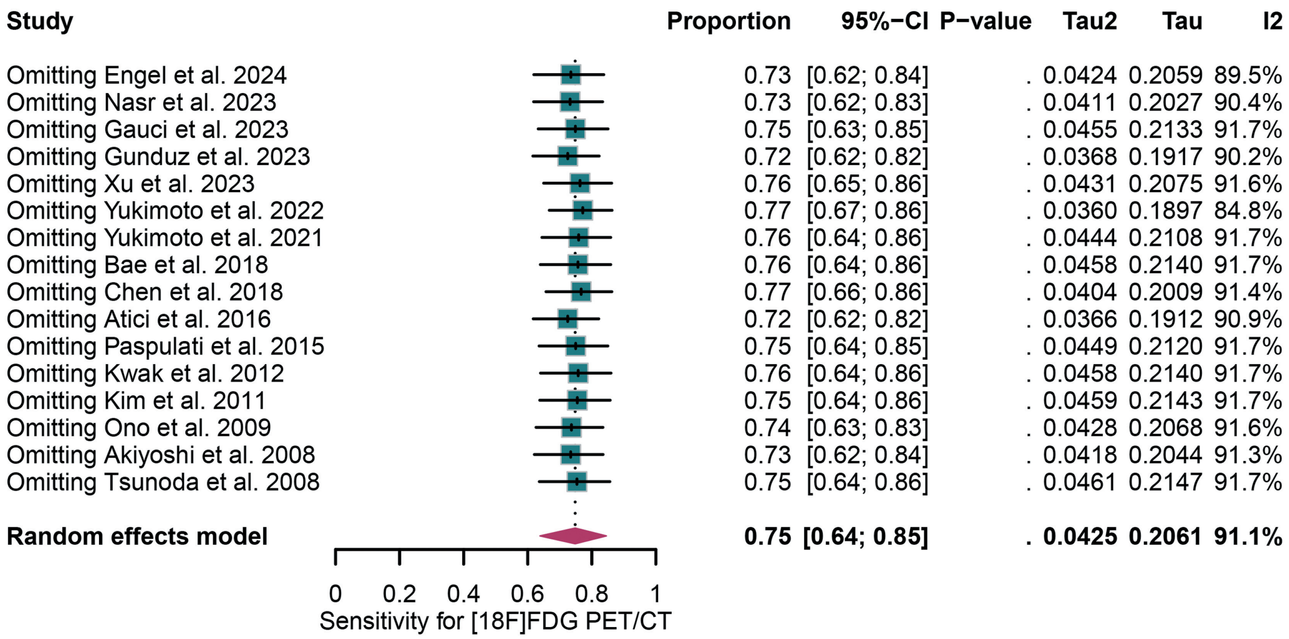

Supplementary Fig. S1 Leave-one-out sensitivity analysis in sensitivity for [18F]FDG PET/CT.

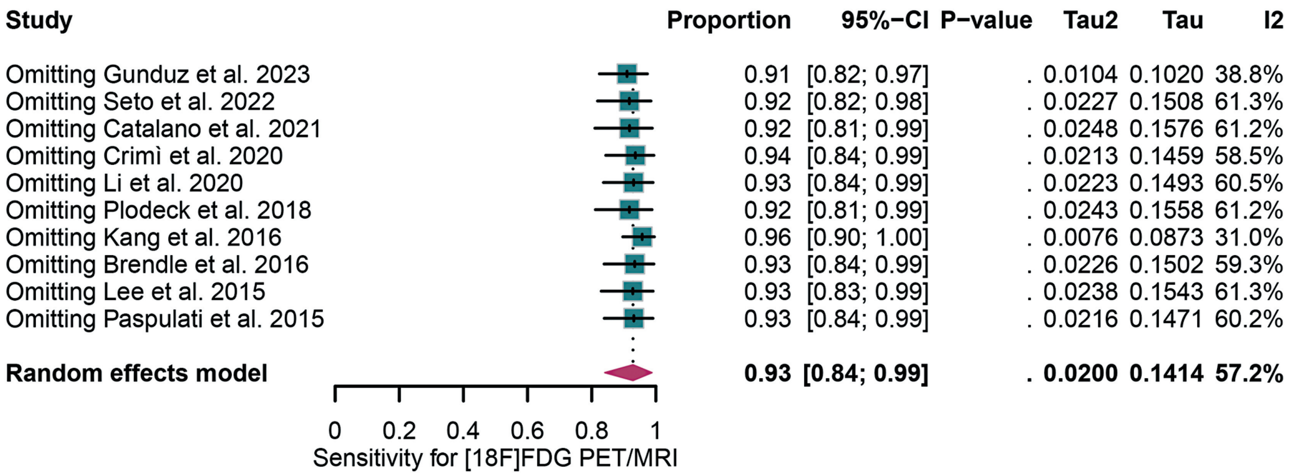

Supplementary Fig. S2 Leave-one-out sensitivity analysis in sensitivity for [18F]FDG PET/MRI.

 © 2025. The Author(s).

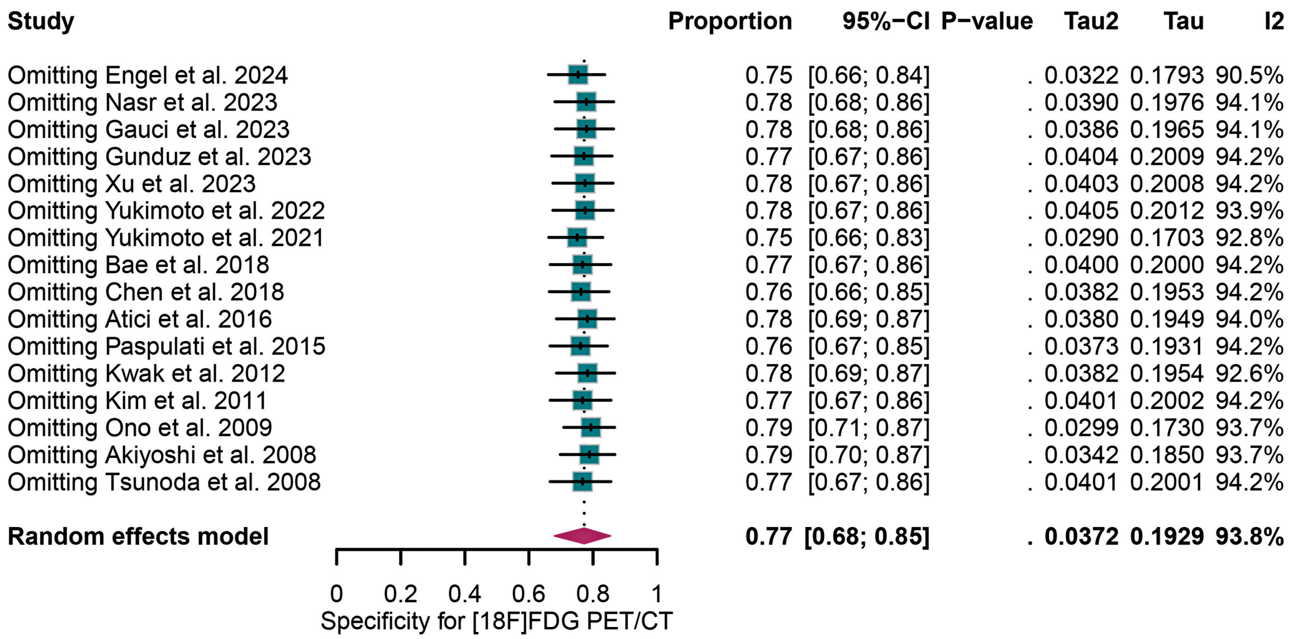

Supplementary Fig. S3 Leave-one-out sensitivity analysis in specificity for [18F]FDG PET/CT.

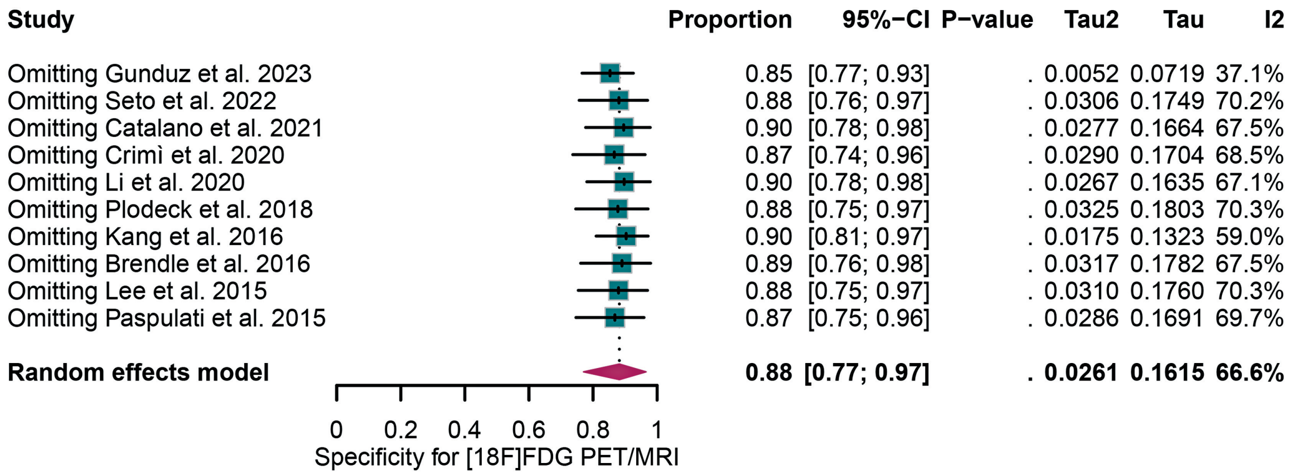

Supplementary Fig. S4 Leave-one-out sensitivity analysis in specificity for [18F]FDG PET/MRI.

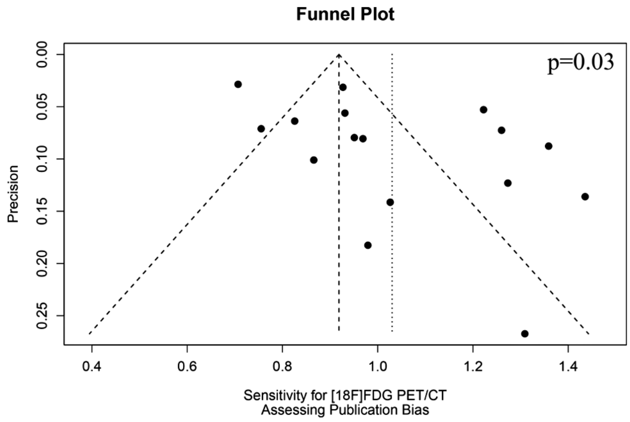

Supplementary Fig. S5 Egger's test in sensitivity for [18F]FDG PET/CT. Funnel plot of studies included for DFS in the meta-analysis. Funnel plot with pseudo 95% confidence limits for assessment of publication bias included in the meta-analysis. The Egger's test revealed that the likelihood of publication bias was low ($p = 0.03$). DFS, disease-free survival.

 © 2025. The Author(s).

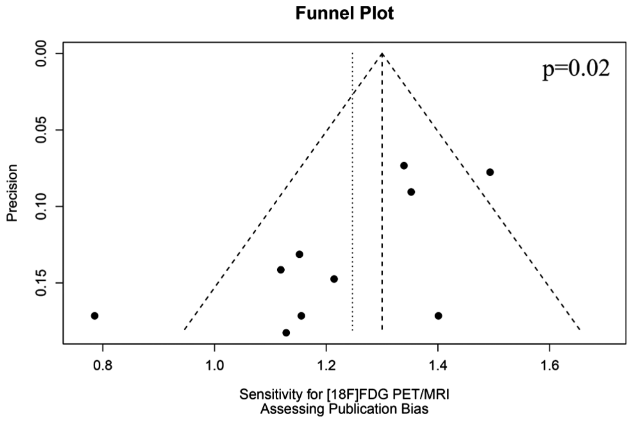

Supplementary Fig. S6 Egger's test in sensitivity for [18F]FDG PET/MRI. Funnel plot of studies included for DFS in the meta-analysis. Funnel plot with pseudo 95% confidence limits for assessment of publication bias included in the meta-analysis. The Egger's test revealed that the likelihood of publication bias was low ($p = 0.02$). DFS, disease-free survival.

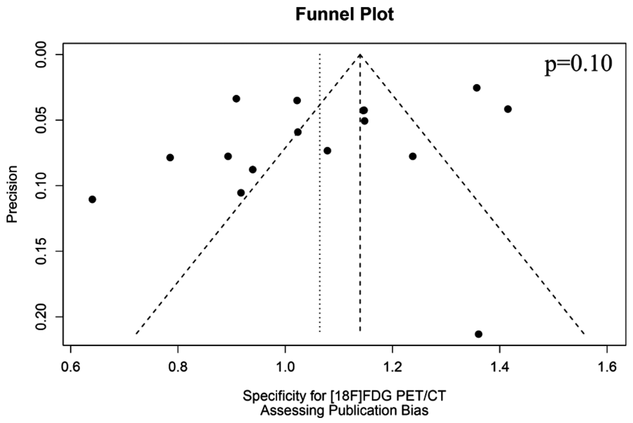

Supplementary Fig. S7 Egger's test in specificity for [18F]FDG PET/CT. Funnel plot of studies included for DFS in the meta-analysis. Funnel plot with pseudo 95% confidence limits for assessment of publication bias included in the meta-analysis. The Egger's test revealed that the likelihood of publication bias was low ($p = 0.10$). DFS, disease-free survival.

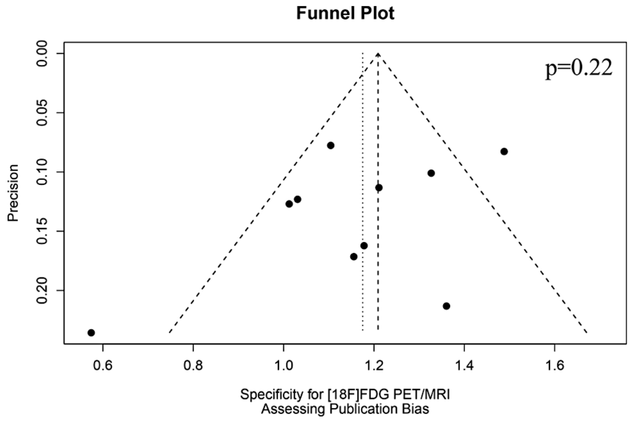

Supplementary Fig. S8 Egger's test in specificity for [18F]FDG PET/MRI. Funnel plot of studies included for DFS in the meta-analysis. Funnel plot with pseudo 95% confidence limits for assessment of publication bias included in the meta-analysis. The Egger's test revealed that the likelihood of publication bias was low ($p = 0.22$). DFS, disease-free survival.
